# Shredder species identity over diversity: Insights into litter decomposition in ponds

**DOI:** 10.1371/journal.pone.0327999

**Published:** 2025-08-14

**Authors:** Emília Židišinová, Milan Novikmec, Marek Svitok

**Affiliations:** 1 Department of Biology and General Ecology, Faculty of Ecology and Environmental Sciences, Technical University in Zvolen, Zvolen, Slovakia; 2 Department of Forest Ecology, Faculty of Forestry and Wood Sciences, Czech University of Life Sciences Prague, Prague, Czech Republic; 3 Institute of Botany, Plant Science and Biodiversity Centre, Slovak Academy of Sciences, Bratislava, Slovakia; King's College London, UNITED KINGDOM OF GREAT BRITAIN AND NORTHERN IRELAND

## Abstract

Many freshwater ecosystems rely on the decomposition of organic matter as a key process for nutrient cycling and energy flow. Small lentic freshwater ecosystems, such as ponds, often derive a large amount of energy from allochthonous detritus due to their close connection with the terrestrial environment. However, the process of leaf litter decomposition in ponds remains poorly understood. We conducted a microcosm experiment in a pond environment to investigate intra- and inter-specific variation in organic matter processing by three shredders (*Tipula* sp., *Sericostoma* sp. and *Gammarus fossarum*) and to assess the effects of shredder community characteristics on the mass loss of black alder (*Alnus glutinosa*) leaf litter. We developed a novel approach to quantify functional traits directly related to litter processing. Detailed gut content analysis revealed significant inter- and intra-specific variation in the organic matter particles ingested by individual shredder taxa. Our results showed that neither taxonomic nor functional diversity reliably predicts leaf litter decomposition rates in ponds. Instead, the keystone shredder *Sericostoma* showed a pronounced effect on decomposition rates driven by their unique feeding behaviour and density-dependent shifts in particle size preferences. These findings highlight the importance of a detailed understanding of species-specific functional traits and behaviour in shaping ecosystem processes, as the role of keystone species can outweigh the contributions of overall diversity measures in driving ecosystem processes.

## Introduction

The decomposition of organic matter plays a key role in the carbon cycle and nutrient dynamics of ecosystems [1]. For many freshwater food webs, the organic matter from allochthonous leaf litter is the primary energy source [2,3], including ponds [4–6], and its decomposition is a key ecological process affecting aquatic ecosystem functioning [3,7]. Leaf litter decomposition is controlled by many interacting physical, chemical and biological factors [1,8]. The diversity of invertebrates consuming coarse organic matter (shredders) is often considered an important component in this process and is frequently studied, primarily in terms of taxonomic richness [1,9]. Previous research has shown that shredder species richness is an important factor in leaf litter breakdown [e.g., 10,11].

However organisms with different phylogenies can be functionally similar, occupying the same ecological niches [12–15]. This functional convergence suggests that relying solely on taxonomic measures in studies of diversity effects on ecosystem functioning may be insufficient, as it may overlook functional attributes that drive ecosystem processes [e.g., 16,17]. To understand how biodiversity is related to ecosystem functioning, functional diversity is often evoked as a promising tool to mechanistically link those components [18].

Functional diversity is a biodiversity measure that represents a set of species traits affecting the ecosystem function of interest [19,20] and has an important role in leaf litter decomposition in freshwater ecosystems [e.g., 17,21,22]. Considering organic matter processing, functional diversity may be expressed through a variety of functional traits [20] that are related to invertebrate feeding activity. Aquatic ecologists have traditionally used the classification that connects morphological characteristics of invertebrates (e.g., mouthparts specialization) and their behavioural mechanisms (e.g., feeding mode) in the processing of food resources (functional feeding groups) [23,24]. However, the classification into trophic guilds is relatively broad and does not allow for a fine differentiation of feeding mode among species within the same group [25–27]. These inter-specific differences can have a significant influence on organic matter processing. For example, Jonsson & Malmqvist [28] considering leaf litter breakdown and shredder identity note that species within the same trophic group may not be redundant as they influenced the decomposition rate differently. Obviously, linking shredder diversity to leaf litter breakdown requires evaluating functional diversity based on species traits directly related to food processing [e.g., 29,30].

Within the same feeding guild, different shredder species may vary in their methods of leaf litter processing, making them functionally distinct. For instance, Graça et al. [31] observed that *Asellus aquaticus* feeds by scraping the leaf surface, whereas *Gammarus pulex* nibbles the leaf. Some shredders leave roughened and frayed leaf surfaces with many holes, facilitating consumption by other organisms [32]. Tonin et al. [22] found that *Sericostoma* caddisflies shred whole litter discs, including less palatable parts, while *Lepidostoma* consumes only the margins and, during feeding, releases high amounts of smaller litter fragments that could be used by other species. Thus, smaller and less effective shredders can benefit from the specific feeding methods of larger ones and complement them in the litter processing process. However, the extent to which feeding mode affects litter processing remains unclear.

Moreover, several studies have shown that the presence of a particular shredder species can have a disproportionately large effect on litter breakdown rates [e.g., 33–36], suggesting that the presence of specific traits, rather than changes in overall shredder diversity, can be the key driver of the litter decomposition process. There is a need for research considering the importance of functionally dominant traits in shredder communities rather than merely species richness effects on litter decomposition [37].

The relationship between organic matter breakdown and shredder functional diversity in freshwater ecosystems has received little attention [13] and existing studies have focused almost exclusively on running waters [21,38, but see 17]. Although leaf litter can provide important energy subsidies to lentic ecosystems [39,40] only a few studies have explored litter breakdown in ponds that are tightly connected to the terrestrial environment [4,17,41–43].

In the present study, using detailed gut-content analysis, we measured several functional traits that are directly related to leaf litter processing, and we evaluated the effect of taxonomic and functional diversity of shredders on leaf litter breakdown in a field experiment conducted in a pond. Our aims were: 1) to compare intra- and inter-specific variation in leaf litter processing by shredders, and 2) to assess the effect of taxonomic diversity, functional diversity and species identity on leaf litter mass loss. We hypothesized that functional diversity is more closely related to ecosystem functioning than taxonomic diversity. We also assumed that individual shredder species are not functionally equivalent but occupy different positions in functional trait space encompassing leaf litter processing and feeding modes.

## Materials and methods

### Study site

The field experiment was conducted in a pond located in the Malá Fatra Mts., Central Slovakia [49°05´54.7ʺN; 18°45´31.0ʺE; 752 m asl]. It is a small artificial pond with an area of 172 m^2^ and an average depth of 0.84 m with substrates composed of sand (70%), fine sediment (20%), and gravel (10%) bottom. The riparian zone is partly covered by trees (*Alnus glutinosa, Salix* sp. and *Picea abies*). Dominant aquatic macrophytes are *Potamogeton natans* and *Lemna minor*. The pond is fed by rainwater and a nearby brook. The aquatic environment has low nutrient content (nitrate: 3.11 mg/L, orthophosphate: 0.02 mg/L), a circumneutral pH (7.3) and relatively low conductivity (120 µS/cm).

### Field experiment

We conducted a microcosm experiment in the autumn of 2018 (September–October) to examine inter-specific variation in leaf litter processing by shredders (Aim 1) and to assess the effects of shredder diversity on the mass loss of black alder (*Alnus glutinosa*) leaf litter in a pond environment (Aim 2). Microcosms were made of acrylic cylinders (diameter 7.3 cm, height 11.7 cm) covered with 250 µm nylon mesh at the top and bottom to allow the passage of water and microorganisms but to prevent the entry of macroinvertebrates. Leaves of black alder were chosen because the species is common in native riparian vegetation in the area and its leaves are typical for fast microbial conditioning, have high N content and low content of lignin, cellulose and recalcitrant compounds [44,45].

The leaves were collected from trees one day before the experiment. We decided to use fresh leaves to prevent bias related to drying [7] and because shredders often prefer fresh over dry leaves [46, own observation]. We gently removed all leaf petioles and weighed groups of three approximately equal-sized leaves to the nearest 0.01 mg. The pre-weighed leaf groups were placed into the microcosms for the first 33 days without shredders to allow for microbial conditioning, which makes the leaves more palatable and nutritionally rich for shredders [47–49]. The selected conditioning period of approximately one month allows thorough colonization by aquatic fungi and microbes, as several studies have shown that fungal biomass on black alder leaves reach high values at around 30 days of immersion and is strongly positively correlated with shredder-mediated breakdown and consumption rates [e.g., 50,51]. Three shredder taxa were used in the experiment: *Tipula* sp. (Diptera), *Sericostoma* sp. (Trichoptera), and *Gammarus fossarum* (Crustacea) (*Tipula*, *Sericostoma,* and *Gammarus*, respectively, hereafter). Most shredder individuals were collected from the study pond, supplemented by a few individuals captured from a nearby brook which is hydrologically connected to the study pond. Shredders were sampled just before the experiment began and identified using identification keys by Jażdżewski [52], Hofsvang [53] and Waringer & Graf [54]. We directly manipulated taxonomic diversity (1–3 shredder taxa per microcosm) and indirectly also functional diversity (through different species combinations for each level of taxonomic diversity), while maintaining a constant total density of 6 individuals per microcosm (S1 Table). Shredder specimens were introduced into the microcosms in a crossed factorial design with three replicates of single-species microcosms (3 × *Gammarus*, 3 × *Sericostoma*, 3 × *Tipula*), three replicates of two-species mixture microcosms (3 × *Gammarus* and *Sericostoma*, 3 × *Gammarus* and *Tipula,* 3 × *Sericostoma* and *Tipula*) and nine replicates of three-species microcosms (9 × *Gammarus*, *Sericostoma* and *Tipula*), resulting in a total of 27 microcosms and 162 individuals (54 specimens per taxon). A higher number of three-species microcosms was established to increase the precision of effect estimates, as we expected greater heterogeneity in species-rich assemblages due to potential interspecific interactions. All microcosms were randomly placed in plastic baskets and positioned near the pond bank. We did not fully submerge the microcosms, leaving approximately 1.5 cm of the upper parts above the water surface to allow the larvae of *Tipula* access to atmospheric oxygen. The pre-conditioned leaves were exposed to the shredders for 7 days. Afterwards, the leaves from each microcosm were placed in plastic bags and stored in a portable freezer at −20°C. The shredders were preserved in 70% ethanol in the field and, along with the collected leaves, were transported to the laboratory on the same day for further processing.

### Laboratory analysis

The leaf samples were processed immediately upon their arrival at the laboratory following the protocol of Benfield et al. [8]. Briefly, the leaves were gently cleaned of organic deposits and other dirt and subsequently dried for 24 h at 50 °C, weighed and combusted in a muffle furnace for 3 h at 550 °C. The ash was wetted with distilled water and dried again for 48 h at 50 °C. After this process, we calculated ash-free dry mass (AFDM). In the same way, we calculated the proportion of AFDM in set-aside leaf samples that were not used in the microcosms. This proportion of AFDM in fresh leaves was used to estimate the initial AFDM of the leaves that were used in the experiments. The loss of organic matter in the microcosms was quantified as the proportion of AFDM lost, calculated by subtracting the ratio of remaining AFDM to initial AFDM from 1.

We measured the body length of all individuals using a binocular microscope equipped with a micrometric eyepiece. For caddisflies, the total body length was measured from the tip of the head to the end of the abdomen, while for dipterans, the measurement was taken from the uppermost part of the body to the end of the abdomen. For body length measurements of *Gammarus*, we used a technique described by Asochakov [55]. To explore intra- and inter-specific variation in litter processing among shredders, we analysed the gut contents of each individual using a technique modified by Felten et al. [56]. Shredder specimens were dissected using tweezers, and the material from the anterior part of their digestive tract was homogenized in a droplet of distilled water and subsequently mounted on slides for gut content analysis. Only the foregut content was analyzed to minimize the effects of digestion on ingested particles.

For each slide, 4–6 non-overlapping fields of view were imaged at 600 × magnification using an Olympus BX40 microscope equipped with the Quick Photo software. The initial field of view was selected at random and subsequent images were taken by moving the stage in randomly selected intervals of 1–5 screw turns in both X and Y directions to avoid overlap and ensure representative spatial coverage. In all the pictures, ingested leaf particles were counted, and their sizes were estimated in ImageJ v. 1.52a [57]. Prior to the analysis, pictures were pre-processed removing all larger non-leaf material (mainly animal tissues and amorphous detritus). We set a lower limit of the particle size to 100 µm^2^ which allowed us to separate fine detritus from the leaf particles.

### Shredder community characteristics

The shredder community in each microcosm was characterized by its taxonomic and functional diversity, as well as by qualitative (presence/absence) and quantitative (density) species composition. Taxonomic diversity was quantified as the number of species present in each microcosm (species richness). To calculate functional diversity, we compiled a matrix of functional traits. There is ongoing debate about the number and type of traits to include in a trait matrix for calculating functional diversity [13,58]. Direct measurements are generally considered more accurate for defining species traits than database-derived values [58]. Because food particle information is rarely available, we used particle characteristics measured for each individual (S2 Table) as a baseline to calculate four traits directly related to leaf litter processing: minimum particle size, maximum particle size, mean particle size, and particle size variability (coefficient of variation). We also included body size and voltinism which has been shown as a crucial functional trait mediating the effects of shredder diversity on litter decomposition [16,17]. Voltinism was treated as a continuous trait representing the number of generations per year, with values assigned as 0.5 for semivoltine *Sericostoma*, 1 for univoltine *Tipula* and 3 for polyvoltine *Gammarus*, following Šporka et al. [59]. Rao’s quadratic entropy (Rao’s Q) [60] was used to calculate functional diversity. Rao’s Q is the most widely used functional diversity measure [13] representing the mean functional dissimilarity between two randomly chosen individuals [61].

#### Ethics statement.

No permits were required for research and field collection of benthic organisms at the study site.

### Data analysis

We assessed variation in leaf litter processing among shredder species (Aim 1) by examining the overlap in ingested particle size distributions and testing for differences in mean ingested particle size. Kernel density estimation was used to approximate the distribution of particle sizes for each species. Subsequently, the degree of similarity in the size distribution of leaf particles among species was quantified using the integrals of the minima of overlapping densities [62].

We used a linear mixed-effects model (LMM) [63] to compare mean particle sizes among shredder species. Mean particle sizes were modelled as a function of species identity (three levels: *Gammarus*, *Sericostoma* and *Tipula*) and body length to account for the allometric relationship with body size [64]. Individual microcosms, nested shredder individuals and multiple microscope fields of view per shredder were included as random effects in the LMM to reflect the hierarchical structure of our sampling design. Model performance was evaluated through a simulation-based approach to residual diagnostics [65]. To enhance the symmetry of the distribution and stabilize variances, particle size data were logarithmically transformed. Following model refitting, no considerable violations of the model assumptions were detected. We evaluated statistical significance using parametric bootstrap tests (10,000 resamples), a robust and flexible method that allows testing of both fixed and random effects and performs well in small-sample studies [66,67]. The overall test was followed by Tukey-type contrasts to assess pairwise differences among individual species [68]. We calculated semi-partial marginal coefficients of determination (R^2^_m_) and intra-class correlation coefficients (ICC) to quantify the variance explained by individual fixed and random effects [69].

LMMs were used to gain further insight into the variation in ingested particle sizes within the shredder species. Three species-specific models were fitted following the procedure outlined above. We explored the effect of species density (1–6 individuals of a given species per microcosm) on ingested particle size while accounting for body length and the hierarchical structure of the sampling design.

A series of beta regression models [70] was fitted to evaluate the effects of shredder identity and diversity on the leaf litter mass loss (Aim 2). This approach is advantageous when modelling variables with values bounded within a standard unit interval, such as relative litter mass loss, which is restricted to the range of 0–100%. We modelled mass loss as a logit function of shredder community characteristics (taxonomic diversity, functional diversity, species identity and species-specific density) assuming that mass loss follows a beta probability distribution defined by mean and precision parameters [71]. Because the community characteristics are mutually correlated, their effects were examined in separate models. The models of species identity and species-specific densities also included interaction effects of species presences and densities, respectively, to evaluate the potential influence of inter-specific relationships such as competition and facilitation [22]. We kept the precision parameter constant, corresponding to the basic beta regression model [70]. The model parameters were estimated by maximum likelihood and goodness-of-fit was assessed using diagnostic plots. Species-specific densities were square-root transformed to improve the convergence of the beta regression models. Since one of the microcosms emerged as an outlier in all models (generalized leverage > 100 and absolute standardized weighted residuals 2.5‒6), we excluded this microcosm, which had exceptionally low litter mass loss (<1% AFDM), from the analysis. Refitting the models without this outlier substantially improved the regression diagnostics. Nevertheless, we also report the results of the full models including the outlier (S3 Table), which show that the same overall trends emerged with and without its inclusion, although p-values increased slightly after excluding the outlier. The significance of model coefficients was evaluated using partial Wald tests [71].

All analyses were performed in R v. 4.2.0 [72] using the libraries betareg [71], DHARMa [73], emmeans [74], ggplot2 [75], ggridges [76], lme4 [77], lmerTest [78], overlapping [79], performance [80] and vegan [81].

## Results

### Differences in leaf litter particle size among and within species

**Fig 1:**
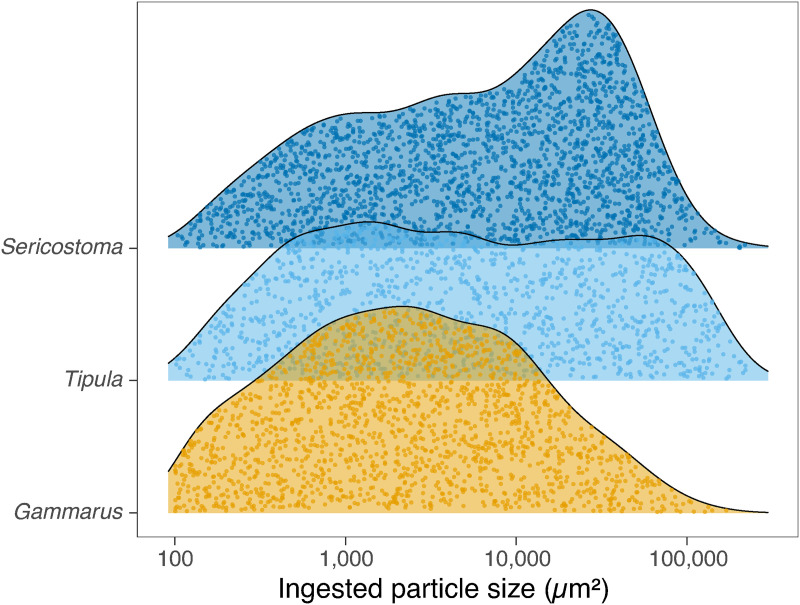
Differences in the size distribution of leaf litter particles ingested by *Sericostoma*, *Tipula* and *Gammarus* represented as Gaussian kernel density estimates. Note that we set a lower limit of the particle size to 100 µm^2^ to separate fine detritus.

The shredder species exhibited distinct patterns in the distribution of ingested particle sizes (Fig 1). *Sericostoma* primarily ingested larger particles (mean ± CV [min, max] = 17608 ± 154 [104, 600186] mm^2^), as indicated by its density peak at the higher end of the particle size range. In contrast, *Gammarus* ingested rather smaller particles (7638 ± 194 [100, 170358] mm^2^), while *Tipula* consumed a broader range of particle sizes without a clear preference (23720 ± 169 [102, 326314] mm^2^). These differences in feeding strategies correspond to the degree of overlap among kernel density estimates. The lowest overlap was observed between *Gammarus* and *Sericostoma* (75%), followed by *Gammarus* and *Tipula* (79%) with *Tipula* and *Sericostoma* showing the highest similarity (84%).

**Fig 2:**
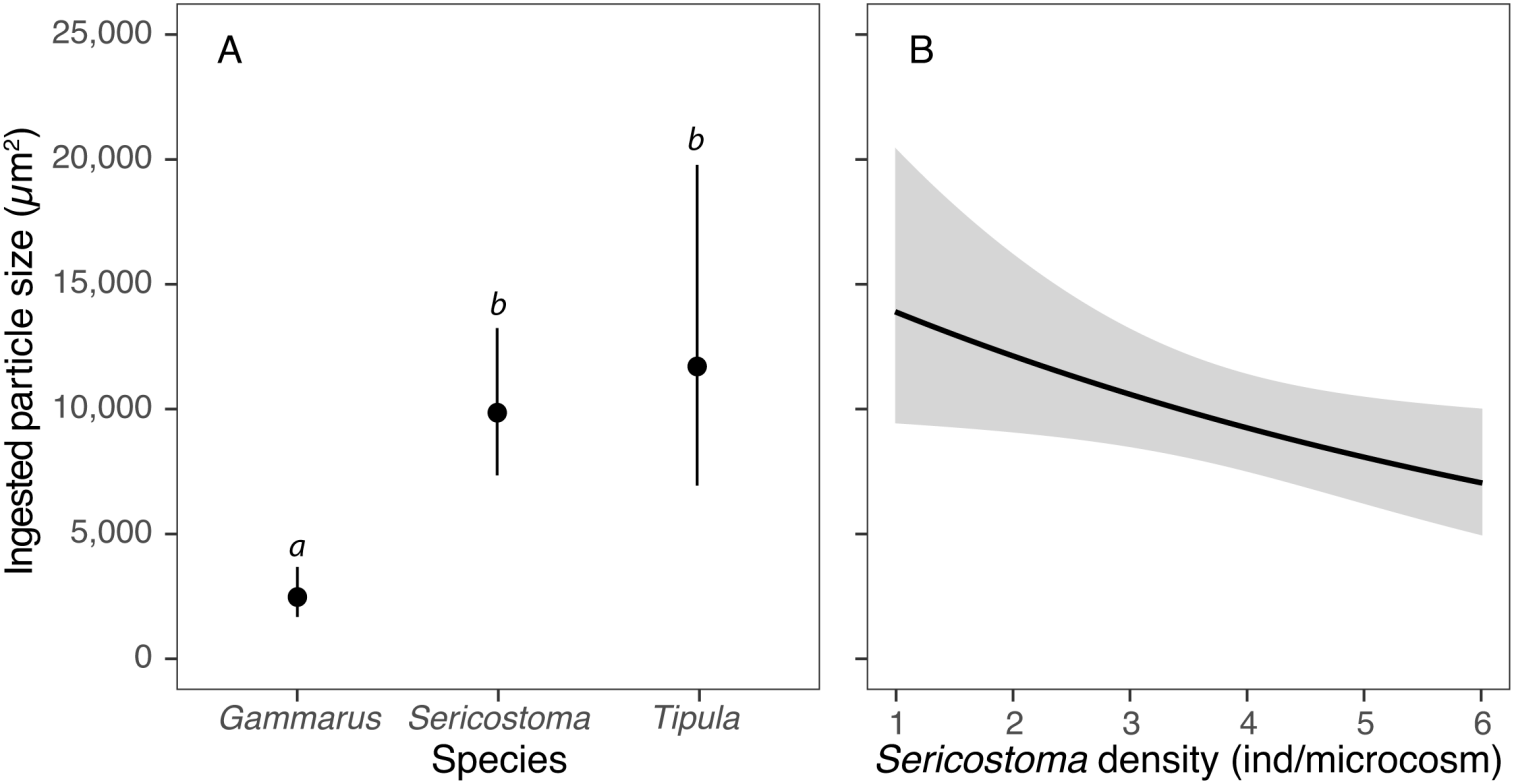
Differences in the mean size of leaf litter particles ingested by *Sericostoma*, *Tipula* and *Gammarus* (A) and the effect of *Sericostoma* density on particle sizes ingested by this species (B). Linear mixed-effects model estimates (dots, lines) are displayed along with their 95% confidence intervals (error bars, grey band). Statistically significant differences between species, as revealed by Tukey contrasts (p < 0.05), are indicated by different italicized lowercase letters.

Inter-specific variation in feeding strategies was further supported by mixed-effects modelling, which revealed significant differences in mean ingested particle sizes among species (Table 1). After accounting for body length, *Gammarus* ingested significantly smaller particles than *Sericostoma* (z = −6.84, P < 0.001) and *Tipula* (z = −3.97, P < 0.001), while mean particle sizes of the latter two species were statistically comparable (z = −0.53, P = 0.86) (Fig 2A). After accounting for fixed effects, significant residual variation was attributable to microcosms (5.9%), individuals nested within microcosms (13.8%) and fields of view nested within individuals (7.1%).

**Table 1:** Results of linear mixed-effects models testing the effects of interspecific differences and density-dependent intraspecific variability on the size of consumed leaf litter particles.

Model/Parameters	b/σ^2^ [95%CI]	χ^2^/t	P	R^2^_m_/ICC (%)
Inter-specific differences				10.7
**Species**		**38.8**	**<0.001**	**9.0**
***Gammarus* (Intercept)**	**8.28 [7.67, 8.96]**	**25.1**	**<0.001**	**‒**
***Sericostoma***	**1.36 [0.97, 1.78]**	**6.8**	**<0.001**	**‒**
***Tipula***	**1.50 [0.77, 2.31]**	**4.0**	**<0.001**	**‒**
Body length	−0.03 [−0.09, 0.02]	1.4	0.269	0.4
**Microcosm**	**0.17 [0.02, 0.41]**	**5.8**	**0.013**	**5.9**
**Individual**	**0.41 [0.27, 0.59]**	**106**	**0.002**	**13.8**
**Field of view**	**0.21 [0.15, 0.29]**	**103**	**0.001**	**7.1**
Intra-specific variability in *Gammarus*				0.9
Density	0.10 [−0.15, 0.35]	0.72	0.451	0.7
Body length	0.05 [−0.17, 0.24]	0.20	0.665	0.2
Microcosm	0.41 [0.00, 1.02]	2.3	0.058	14.9
**Individual**	**0.30 [0.10, 0.80]**	**17.2**	**0.001**	**11.1**
**Field of view**	**0.21 [0.12, 0.34]**	**46.9**	**<0.001**	**7.7**
Intra-specific variability in *Sericostoma*				1.7
**Density**	**−0.14 [−0.26, −0.02]**	**4.8**	**0.043**	**1.7**
Body length	0.02 [−1.10, 1.15]	0.2	0.664	<0.01
Microcosm	0.03 [0.00, 0.16]	0.02	0.537	1.0
**Individual**	**0.32 [0.17, 0.53]**	**42.1**	**<0.001**	**11.7**
**Field of view**	**0.19 [0.10, 0.30]**	**42.3**	**<0.001**	**6.8**
Intra-specific variability in *Tipula*				2.0
Density	−0.16 [−0.45, 0.14]	1.23	0.358	2.0
Body length	−0.01 [−0.08, 0.06]	0.05	0.849	<0.1
**Microcosm**	**0.50 [0.06, 1.19]**	**5.55**	**0.010**	**13.9**
**Individual**	**0.31 [0.08, 0.76]**	**11.6**	**0.002**	**8.5**
**Field of view**	**0.25 [0.10, 0.47]**	**17.5**	**0.002**	**6.8**

Fixed effect coefficients (b) and random effect variance components (σ^2^) are presented alongside their 95% confidence intervals (CI), test statistics (χ^2^/t), probabilities (P). Semi-partial marginal coefficients of determination (R^2^_m_) and intra-class correlation coefficients (ICC) are reported for fixed and random effects, respectively. Overall R^2^_m_ is also provided for each model (highlighted with a gray background). Results significant at α = 0.05 are shown in bold.

When comparing inter-specific variation, we found that the mean size of particles ingested by *Gammarus* and *Tipula* did not vary significantly with density of these species. In contrast, *Sericostoma* showed a clear response to changes in its density: as density increased, the size of particles ingested decreased, indicating a negative density-dependent pattern (Fig 2B).

### The role of shredder identity, diversity and density in litter decomposition

**Fig 3:**
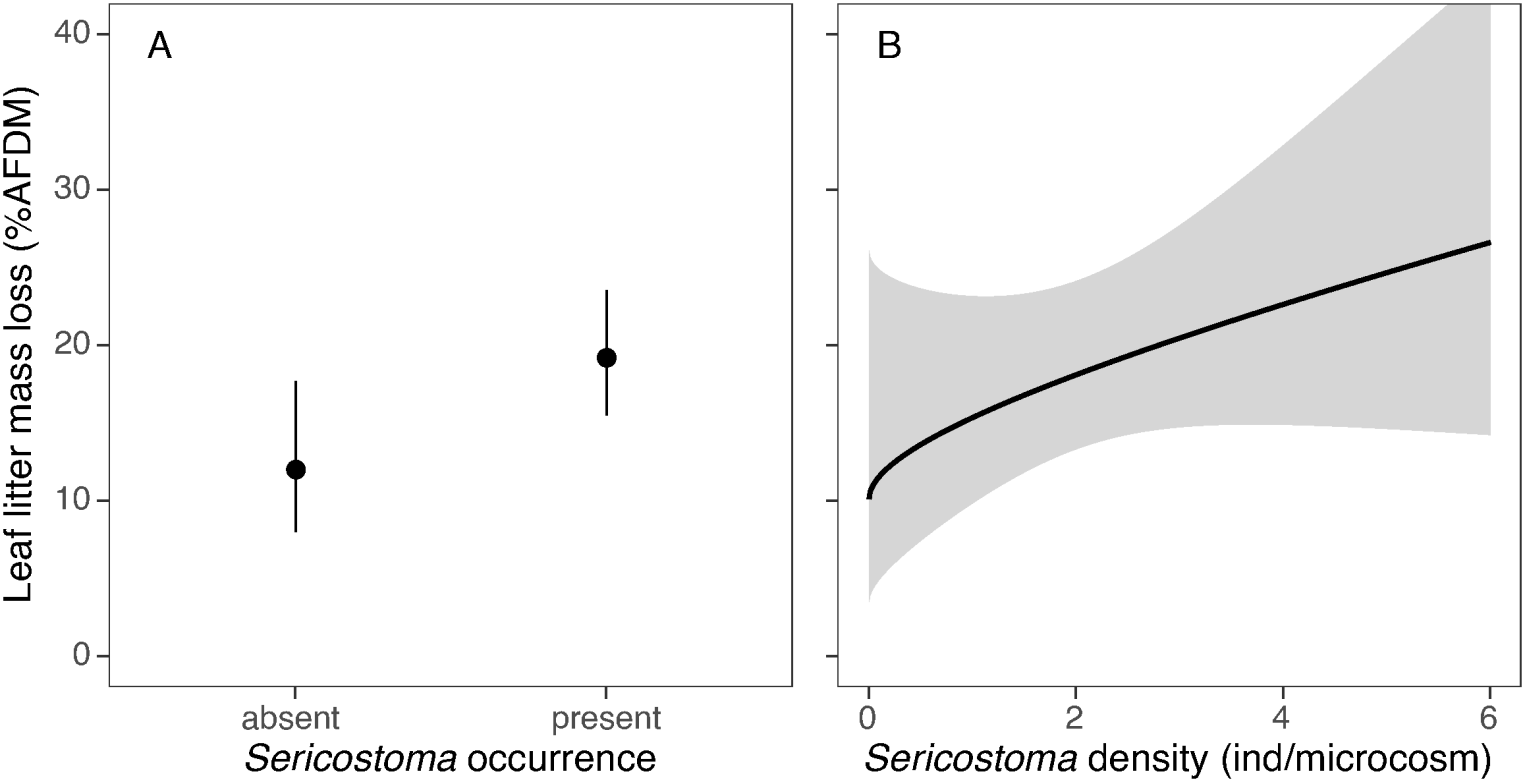
Significant effect of *Sericostoma* presence (A) and density (B) on leaf litter mass loss in a pond environment. Estimates of beta regression models (dots, lines) are displayed along with their 95% confidence intervals (error bars, grey band).

Mean leaf litter mass loss ranged from 12.7 to 17.3 and 18.7% AFDM in single-species, two-species and three-species microcosms, respectively (S1 Table). However, neither shredder taxonomic diversity nor functional diversity had a significant effect on leaf litter decomposition in pond microcosms (Table 2). In contrast, species identity mattered, and microcosms with the presence of *Sericostoma* showed significantly faster decomposition than those without this caddisfly (Fig 3A). The identity effect of the other species was indistinguishable from the average contribution. These results were further corroborated by the model involving species-specific densities, which showed a significant positive effect of *Sericostoma* density on litter decomposition (Fig 3B). The density effects of the remaining species were non-significant.

**Table 2:** Results of beta regression models testing the effects of taxonomic diversity, functional diversity, species identity and species-specific density on the leaf litter mass loss.

Model/Parameters	b [95% CI]	χ^2^	P	R^2^ (%)
Taxonomic diversity				2.8
Species richness	0.12 [−0.17, 0.41]	0.68	0.410	
Functional diversity				0.01
Rao’s Q	−0.16 [−6.80, 6.45]	<0.01	0.962	
Species identity				19.5
*Gammarus*	0.34 [−1.24, 1.93]	0.50	0.480	1.1
*Tipula*	0.20 [−1.29, 1.69]	1.19	0.276	2.3
***Sericostoma***	**0.52 [−1.07, 2.12]**	**4.63**	**0.032**	**13.8**
*Gammarus × Tipula*	−0.48 [−1.59, 0.64]	0.70	0.403	2.0
*Tipula × Sericostoma*	−0.14 [−1.34, 1.05]	0.06	0.812	0.1
*Gammarus × Sericostoma*	−0.18 [−1.15, 1.52]	0.07	0.788	0.03
Species density				19.5
*Gammarus*	0.61 [−1.24, 1.93]	0.84	0.358	2.7
*Tipula*	0.55 [−1.29, 1.69]	0.32	0.573	0.05
***Sericostoma***	**0.69 [−1.07, 2.12]**	**4.25**	**0.039**	**9.9**
*Gammarus × Tipula*	−0.27 [−1.59, 0.64]	0.21	0.651	0.6
*Tipula × Sericostoma*	−0.14 [−1.34, 1.05]	0.05	0.819	0.1
*Gammarus × Sericostoma*	−0.01 [−1.15, 1.52]	<0.01	0.982	<0.01

Model coefficients (b) are presented alongside their standard 95% confidence intervals (CI), test statistics (χ^2^) and probabilities (P). Pseudo-determination coefficients (R^2^) of the full models (highlighted in gray) and semi-partial R^2^ values for individual effects are reported. Results significant at α = 0.05 are highlighted in bold. For the results of models based on the full dataset, including an outlying observation, see S3 Table.

In the analysis, we excluded an outlying mesocosm with very low litter loss (<1% AFDM), as its inclusion compromised the assumptions of the models. Keeping the outlier in the dataset would lead to even stronger support for the above conclusions and would reveal an additional positive effect of taxonomic diversity on litter mass loss (S3 Table). However, we preferred to prioritize model robustness and have retained a conservative interpretation based on the reduced dataset.

## Discussion

We found evidence of inter-specific variation in food processing among three shredder species. Although there is limited information on feeding modes among shredder taxa, it is highly unlikely that any two species have identical feeding preferences or strategies [11]. As has been shown in our experiment, after accounting for body sizes, *Gammarus* ingested significantly smaller food particles than *Tipula* and *Sericostoma*, respectively, with substantial variation in particle size distribution observed among the shredder species. Individuals of the amphipod *G. fossarum* typically bite off small particles while larvae of *Tipula* species are known as large particle feeders [82]. Large litter particles were also processed by *Sericostoma* caddisflies, which possess mouthparts highly specialized for fragmenting leaf material, including tougher leaf components [22]. The different feeding strategies of shredders may play a role in leaf litter processing rates. For example, *Tipula* and *Sericostoma*, which bite larger pieces of litter, have been shown to have considerably lower weight-specific leaf litter processing rates compared to *Gammarus*, which bites smaller pieces [29,83]. Smaller particles have a higher surface area relative to their volume, allowing digestive enzymes to access a greater proportion of the material more efficiently, which is advantageous for gammarids with higher metabolic rates compared to *Sericostoma* caddisflies [29].

We also observed intra-specific variation in the size of ingested particles in *Sericostoma* caddisflies with a significant decrease in particle size as the number of individuals increased. The observed consumption of smaller particles at higher conspecific densities may be explained by intra-specific competition, where feeding strategy shifts occur at higher densities [21]. When the density of individuals is higher, competition for food resources increases [28] and the biting off larger pieces of leaves may become more contested, prompting individuals to shift toward smaller, more accessible fragments that are easier to handle and process, facilitating quicker feeding in a competitive environment. Moreover, in a high-density environment, feeding activity might fragment larger pieces into smaller particles, increasing the relative abundance of smaller detritus and making them a more common food source [22]. The observed density-dependent feeding strategy shifts in *Sericostoma* may enhance resource processing rates as density increases (Fig 3B), a behaviour absent for *Gammarus* and *Tipula*, which retain consistent feeding strategies regardless of conspecific densities.

In general, intra-specific variation is increasingly recognized as crucial for understanding ecological processes [84–87]. According to Des Roches et al. [84], intra-specific variation can affect ecological dynamics to the same extent as the removal or replacement of a species in the environment. Similarly, the meta-analysis by Raffard et al. [87] shows that intra-specific variation has significant ecological effects across a large set of species and is involved in shaping ecological dynamics. Rota et al. [83] demonstrated that approximately one-third of the total variation in litter consumption rates among aquatic detritivores can be attributed to variability within species. This suggests that intra-specific variability in traits related to litter consumption can influence ecosystem functions as significantly as inter-specific variation.

We did not find a significant effect of taxonomic diversity on the leaf litter decomposition rate in microcosms in a pond environment. The effects of shredder diversity on decomposition rates are complex and context-dependent. While several studies have found significant positive influences of shredder richness on decomposition [e.g., 28,88,89], others have reported only weak or no effects of shredder taxonomic diversity [e.g., 17,34,35]. These contrasting findings may result from the intricate relationship between the number of species and their functions within ecosystems. Species richness may serve as a suitable surrogate for ecosystem function only under the unlikely assumption of random or uniform niche occupancy [90]. The assembly of pond communities is often non-random, with niches that tend to cluster [17,91]. Therefore, the extent to which an increasing number of species enhances ecosystem process rates depends on their niche complementarity and the addition of new species with similar traits may have minimal effects on ecosystem functioning.

Gessner et al. [1] assumed that direct functional characterization of communities, such as functional diversity, should be a more compelling predictor of aquatic biodiversity effects on litter decomposition than measures of taxonomic diversity. Different feeding strategies, observed in our study, may lower competition and enhance niche complementary among shredding invertebrates, resulting in increased decomposition rates [37]. However, even though we calculated functional diversity based on traits directly linked to feeding activity, the model failed to explain the variation in litter processing rates. Direct comparisons with other studies are challenging, as research on the relationship between functional diversity and litter decomposition in freshwater ecosystems is scarce and yields inconclusive results. For instance, Dekanová et al. [17] observed a significant positive relationship between the functional diversity of shredder communities and organic matter decomposition rates in ponds, emphasizing the importance of body size variability as a key functional trait driving ecosystem processes. In contrast, Frainer et al. [21] reported that the effects of stream invertebrate functional diversity on decomposition fluctuate over time and space, while Voß & Schäfer [38] found no association between invertebrate functional diversity and litter decomposition in low-order streams. A wide range of traits and methods for quantifying functional diversity, along with a general lack of freshwater studies linking functional diversity with ecosystem processes [13], prevent us from drawing more definitive conclusions. However, it seems that community-wide measures of functional diversity may not always capture the complex, context-dependent interactions that drive ecosystem processes.

In our experiment, litter breakdown was not significantly affected by shredder species richness or functional diversity; instead, it was primarily driven by the composition of shredder communities. The presence of the keystone shredder *Sericostoma* had a pronounced effect on the decomposition rate, which further accelerated as its proportion in the species mixture increased. Our finding contradicts the redundancy hypothesis, which assumes that high species abundance can compensate for low species richness, thereby maintaining ecosystem functions as long as all functional groups are represented [92]. The significant intra-guild differences in contributions to decomposition rates align with the idea that even individual species within the same functional feeding group can exhibit substantial variation in their ecological roles [25,26]. An increasing body of research in both terrestrial [93] and aquatic ecosystems [e.g., 33–36] identifies the key role of species identity in litter breakdown. For instance, Encalada et al. [34] observed that most litter decomposition in headwater streams was driven by a single keystone species, while shredder diversity did not contribute significantly. Similarly, Dangles & Malmqvist [33] found that sites dominated by a single shredder species exhibited higher processing rates than those with more evenly distributed species abundances in shredder communities.

Ecosystem processes can be driven by the activity of individual species with specific traits [16] or through inter-specific interactions that surpass the additive contributions of individual species [19,94]. If interspecific relationships, such as competition or facilitation, had occurred, we would expect to observe significant interaction effects between individual species in their influence on mass loss. However, no significant interacting effects of either species occurrences or densities were observed (Table 2). This suggests that the identity effect of *Sericostoma* is primarily driven by its specific traits. Assumed density-dependent feeding plasticity and the ability to process large particles observed in our study, along with the low selectivity of *Sericostoma* species, whose larvae can utilize a broad range of food sources, including highly refractory litter [29,95], could be among the traits that make *Sericostoma* a highly efficient keystone shredder.

In addition, potential density-related feeding shifts (Fig 2B) may also play a role, although this remains to be confirmed with direct behavioral observations. Rather than focusing on community-wide diversity measures, future research should consider the importance of relevant traits and how environmentally induced variations in these traits lead to altered litter decomposition rates.

A potential limitation of our study may arise from small-scale heterogeneity in microbial decomposition among microcosms. Since we did not quantify microbial decomposition in each microcosm, we cannot rule out the possibility that some portion of the observed litter loss was driven by variation in microbial communities across experimental units. Previous studies have reported heterogeneity in microbial communities among replicated litter samples, suggesting potential variation in community composition between microcosms [e.g., 96,97]. However, we do not expect that such stochastic variability significantly biased our results, as community divergence tends to increase over time, and the short duration of our experiment makes it unlikely that early-stage dissimilarities impeded the study of community function [96].

## Conclusion

Our study quantified intra-guild variation in food processing among shredder species and provides insights into the mechanisms underlying shredder contributions to leaf litter decomposition in ponds. Neither the taxonomic nor functional diversity of shredder communities predicted litter mass loss. Instead, the composition of shredder communities, particularly the presence and density of a keystone species *Sericostoma*, emerged as a dominant driver of the decomposition process. The pronounced effect of this shredder is attributable to its specific functional traits, including potential density-dependent feeding plasticity and the ability to process large particles, supporting the notion that species-specific traits and behaviors can outweigh the contributions of overall diversity measures in driving key ecosystem processes. However, additional research on other pond types and shredder communities is required to determine whether our findings can be generalized across diverse ecological contexts.

Since the unique characteristics of individual species can profoundly shape ecosystem processes, the loss of a functionally important species cannot always be compensated for by other species within the same guild and such losses may destabilize ecosystem processes. Therefore, conserving species diversity, even within functional groups, is vital for maintaining ecosystem functioning and resilience. Overall, a detailed understanding of species-specific functional traits is crucial for predicting how changes in biodiversity, such as species loss or shifts in dominance, will influence ecosystem functioning.

## Supporting information

S1 Table Composition and diversity of shedder species assemblages and relative litter loss during the experiment in microcosms. Different assemblages are distinguished by color.
(XLSX)

S2 Table Measured sizes of leaf litter particles ingested by three shredder species in a pond environment. (XLSX)

S3 Table Results of beta regression models testing the effects of taxonomic diversity, functional diversity, species identity and species-specific density on leaf litter mass loss, using the full dataset. This dataset includes an outlying observation with exceptionally low litter AFDM loss (<1%), which resulted in very high standardized residuals (2.5–6) and extreme generalized leverage (>100).
(DOCX)
