# Supplementary material for: Shredder species identity over diversity: Insights into litter decomposition in ponds
Source: PLoS One. 2025 Aug 14;20(8):e0327999. doi: 10.1371/journal.pone.0327999 (PMC12352770; doi:10.1371/journal.pone.0327999)
Supplement: S3 Table — This dataset includes an outlying observation with exceptionally low litter AFDM loss (<1%), which resulted in very high standardized residuals (2.5–6) and extreme generalized leverage (>100). (DOCX) [file pone.0327999.s003.docx]

**Table S3. Results of beta regression models testing the effects of taxonomic diversity, functional diversity, species identity and species-specific density on leaf litter mass loss, using the full dataset. This dataset includes an outlying observation with exceptionally low litter AFDM loss (<1%), which resulted in very high standardized residuals (2.5 – 6) and extreme generalized leverage (>100).**

| Model/Parameters | b [95% CI] | χ^2^ | P | R^2^ (%) |
| --- | --- | --- | --- | --- |
| Taxonomic diversity |  |  |  | 8.0 |
| **Species richness** | **0.42 [0.05, 0.79]** | **4.95** | **0.026** |  |
| Functional diversity |  |  |  | 3.0 |
| Rao's Q | 5.545 [-2.91, 14.0] | 1.65 | 0.199 |  |
| Species identity |  |  |  | 33.5 |
| *Gammarus* | 1.96 [0.14, 3.81] | 2.62 | 0.106 | 8.0 |
| *Tipula* | 0.19 [-1.45, 1.84] | 0.89 | 0.345 | 3.3 |
| ***Sericostoma*** | **2.15 [0.30, 4.00]** | **8.66** | **0.003** | **14.8** |
| *Gammarus × Tipula* | -0.46 [-1.70, 0.78] | 0.53 | 0.465 | 0.3 |
| *Tipula × Sericostoma* | -0.14 [-1.46, 1.17] | 0.05 | 0.828 | 0.05 |
| *Gammarus × Sericostoma* | -1.46 [-3.03, 0.10] | 3.36 | 0.067 | 7.3 |
| Species density |  |  |  | 33.8 |
| *Gammarus* | 3.14 [-3.32, 4.55] | 3.22 | 0.073 | 11.4 |
| *Tipula* | 2.42 [-3.34, 4.44] | 0.47 | 0.492 | 0.03 |
| ***Sericostoma*** | **3.21 [-3.25, 4.62]** | **8.00** | **0.005** | **15.8** |
| *Gammarus × Tipula* | -0.73 [-1.42, 0.88] | 1.25 | 0.264 | 1.5 |
| *Tipula × Sericostoma* | -0.61 [-1.32, 1.05] | 0.82 | 0.364 | 1.2 |
| *Gammarus × Sericostoma* | -0.88 [-1.24, 1.21] | 1.55 | 0.213 | 2.7 |

Model coefficients (b) are presented alongside their standard 95% confidence intervals (CI), test statistics (χ^2^) and probabilities (P). Pseudo-determination coefficients (R^2^) of the full models (highlighted in gray) and semi-partial R^2^ values for individual effects are reported. Results significant at α = 0.05 are highlighted in bold. For the results of models based on the dataset with the outlier excluded, see Table 2.
